# Assessment of Knowledge and Attitudes Related to Food Hygiene Among Food Business Operators in Attica, Greece

**DOI:** 10.7759/cureus.103025

**Published:** 2026-02-05

**Authors:** Elias A Chaidoutis, Olympia Chatzimpyrou, Dimitrios Keramydas, Petros Papalexis, Maria Giannari, Vassiliki Pitiriga, Foteini Koutsiari, Theodoros N Sergentanis, Chara Tzavara, Eirini Thymara, Andreas C Lazaris, Nikolaos Kavantzas

**Affiliations:** 1 First Department of Pathology, Medical School of Athens, National and Kapodistrian University of Athens, Athens, GRC; 2 Department of Microbiology, Medical School of Athens, National and Kapodistrian University of Athens, Athens, GRC; 3 Department of Hygiene and Health Controls, Directorate of Public Health and Environmental Hygiene, Ministry of Health, Athens, GRC; 4 Department of Public Health Policy, School of Public Health, University of West Attica, Athens, GRC; 5 Department of Biostatistics, National and Kapodistrian University of Athens, Athens, GRC

**Keywords:** food business operator, food hygiene, mass catering, public health, training

## Abstract

Food safety is a key public health function, as the catering sector is a frequently cited source of outbreaks. This study evaluated the knowledge and attitudes of food business operators (FBOs) in Attica, Greece, regarding food hygiene. A cross-sectional study was carried out in 522 mass catering establishments located in Attica. Knowledge and attitudes were assessed using a 25-item questionnaire. Statistical analysis included the chi-square test and multiple logistic regression to identify factors associated with adequate knowledge and positive attitudes in the study population.

Questions related to hand hygiene (98.9%, n=516) and pathogen identification (*Salmonella*: 98.1%, n=512) received high scores. However, significant gaps in knowledge were identified regarding the transmission of typhoid fever through food (27.6%, n=144) and the risks associated with raw beef (31%, n=162). Overall, 47.9% (n=250) of participants demonstrated "good knowledge" (score ≥ 18/25), while 52.1% (n=272) had insufficient knowledge. In terms of attitudes, 59.8% (n=312) showed a satisfactory level.

Multivariate analysis revealed that good knowledge was significantly associated with larger company size (≥51 employees: OR=2.68, p=0.020), high educational level (Master's/Doctorate: OR=5.04, p=0.006), and familiarity with Hazard Analysis and Critical Control Points (HACCP)-based systems (OR=0.15 for those who were not familiar, p=0.002). In contrast, participants with the role of owner within the company (OR=0.38, p=0.018) and managers (OR=0.36, p=0.021) were less likely to have good knowledge compared to head chefs. Positive attitudes were strongly associated with previous experience in the food industry (OR=12.96, p=0.018) and official inspections in the last five years (OR=1.75, p=0.022).

Although basic awareness of food hygiene issues is widespread, specific technical knowledge remains inadequate among half of all FBOs. Specific training programs, especially for personnel performing the roles of owner and manager, as well as consistent official controls, are important factors in strengthening food safety culture and protecting public health.

## Introduction

Food safety remains a major public health challenge worldwide, despite consumers' fundamental right to safe and healthy food and the existence of extensive regulatory frameworks [1-5]. Each year, approximately 1 in 10 people worldwide suffer from foodborne diseases, resulting in approximately 420,000 deaths [5-7]. Although the WHO European Region has the lowest global burden, more than 23 million people in Europe fall ill each year, resulting in approximately 5,000 deaths [7].

Foodborne illnesses result from consuming food contaminated with infectious agents such as bacteria, viruses, parasites, or toxins, as well as chemical hazards from environmental sources or from food processing, packaging, transportation, and storage [8,9]. More than 200 diseases are transmitted through food, ranging from mild gastrointestinal illnesses to serious conditions such as kidney failure, liver disease, neurological disorders, and cancer [10-13]. The most common causative agents worldwide include norovirus, *Salmonella enterica*, *Campylobacter* spp., and *Staphylococcus aureus* [14]. In Europe, norovirus alone causes approximately 15 million cases annually, highlighting the critical role of personal hygiene, while non-typhoid infections from *Salmonella* and *Campylobacter* are largely linked to the improper handling of poultry and eggs [7,15,16]. *Listeria monocytogenes* causes listeriosis, a serious illness with an increased risk for pregnant women and other vulnerable groups [7].

Despite comprehensive food safety legislation in the European Union, foodborne diseases continue to be a significant burden on public health. According to the European Centre for Disease Prevention and Control (ECDC) data, in 2023, 5,691 foodborne outbreaks were reported in the European Union, involving 52,127 human cases and 65 deaths. *Salmonella* remained the main causative agent in outbreaks, whereas campylobacteriosis accounted for the highest proportion of confirmed human cases [16]. In Greece, surveillance data from the National Public Health Organization (EODY) show that non-typhoid salmonellosis and *Campylobacter* infection were the most frequently reported foodborne diseases in 2024, while listeriosis had the highest mortality rate [17].

Food contamination can occur at any stage of the food chain, particularly during processing, distribution, and final preparation, often due to inappropriate food handling practices [4,6]. Food businesses, especially establishments that serve food directly to consumers, such as restaurants and mass catering facilities, are often involved in reported outbreaks [18-22]. These environments pose a significant public health problem, as they often serve vulnerable populations such as children, the elderly, pregnant women, and immunocompromised individuals [23-26].

Most foodborne illnesses can be prevented through proper food handling and good hygiene practices (GHP’s) [22,27]. The primary responsibility for food safety resides with the food business operator (FBO), who is legally obliged to ensure that food placed on the market is safe and to implement self-monitoring systems based on the principles of Hazard Analysis and Critical Control Points (HACCP) [4,14,19,28-31].

Training in food hygiene is fundamental to effective risk prevention, improving knowledge, shaping positive attitudes, and compliance with hygiene practices, including the implementation of HACCP [1,4,32-44]. In Greece, formal training in food hygiene is mandatory and is provided by the Hellenic Food Authority (EFET) [40]. However, evidence suggests that knowledge gaps still exist among FBOs, particularly in mass catering establishments [45-48]. Furthermore, food control is a key activity, both in the context of self-control and through official control, to ensure food safety and suitability [49-51]. Parameters such as managerial role, educational level, and professional experience have been shown to influence performance in these environments [52,53].

Given the importance of catering businesses for public health in Attica, the most densely populated region of the country and a major tourist destination [50], the purpose of this study is to assess the level of knowledge and attitudes of FBOs in mass catering establishments in the Attica region while investigating how educational background, training, professional experience, managerial role, and other factors influence compliance with food hygiene rules.

## Materials and methods

Study design

A cross-sectional study was conducted in the Region of Attica, Greece, between July 2023 and November 2024. The study population was FBOs in mass catering establishments, including both full-meal services and snack/light meal services (e.g., cafés, refreshment bars, patisseries, bakeries, and bars), officially registered with local Chambers of Commerce.

Questionnaire development and validation

A structured questionnaire for the collection of research data was adapted from Soares et al. (2012) [54], with official permission. The questionnaire included questions concerning the demographic and socioeconomic characteristics of the participants (e.g., age, gender, and education), as well as their knowledge and attitudes regarding food hygiene. To ensure conceptual equivalence in the Greek context, a process of forward and backward translation was followed. The face and content validity were assessed through a pilot test with n=50 participants, which led to revisions of the questions regarding the risks of *Listeria* and protective clothing to align them with the Codex Alimentarius guidelines [4].

Sampling and sample size

Mass-catering establishments from all areas of Attica (Central, Northern, and Southern Sectors of Athens; Piraeus and Islands; and Eastern and Western Attica) were selected through simple random sampling during the period from July 2023 to November 2024. The required sample size consisted of 522 participants, as determined during the study design phase, accounting for an estimated response rate of 70%. The calculation of the required statistical power was performed via the software G*Power (version 3.1.9.2; Heinrich-Heine-Universität Düsseldorf, Düsseldorf, Germany).

Data collection

In total, we contacted 748 establishments registered in the official register of the Hellenic Statistical Authority (ELSTAT) for mass catering establishments (Nomenclature of Economic Activities (NACE) code 56) [55], resulting in 522 valid responses (response rate 69.8%). Meanwhile, the 50 participants in the pilot phase were excluded from the final analysis. Participants were enlisted through face-to-face interviews or online questionnaires (Google Forms; Google LLC, Mountain View, CA, USA). The Bioethics and Ethics Committee of the Medical School of the National and Kapodistrian University of Athens granted official approval for the study protocol on September 24, 2021. In accordance with the requirements of the committee and the Declaration of Helsinki, all participants provided written, detailed, and informed consent prior to their inclusion. The study strictly adhered to the General Data Protection Regulation (GDPR 2016/679) of the European Parliament and Greek law 4624/2019, ensuring complete anonymity, confidentiality, and protection of personal data throughout the research and publication of the results.

Statistical analysis

Data analysis was conducted using IBM SPSS Statistics for Windows, Version 26 (Released 2018; IBM Corp., Armonk, New York, United States). The variables were first tested for normality via the Kolmogorov-Smirnov criterion. Quantitative data are represented as mean ± standard deviation and medians (interquartile ranges (IQRs)), while qualitative data are shown as absolute and relative frequencies (n, %). Knowledge was deemed "inadequate" for scores under 18 out of 25 (<72%). Logistic regression analysis was used to identify independent factors associated with the level of good knowledge and attitudes toward food hygiene. To determine adjusted odds ratios (ORs) and 95% confidence intervals (CIs) for factors linked to good knowledge and attitude, multiple logistic regression analysis was applied. A p-value of less than 0.05 was considered statistically significant, and all reported p-values are two-tailed.

Sample characteristics

The sample consisted of 522 participants, 57.5% (n=300) of whom worked in a full-service mass catering business. For 19.7% (n=103) of them, the business was in the northern sector of Athens, whereas for 19.5% (n=102), it was in the central sector. The median number of customers was 120 (IQR: 50-250), and the number of employees ranged from 1 to 50 for 90.8% (n=474) of the sample. Additionally, 74.5% (n=389) had undergone an inspection by a public authority in the past five years. Of these inspected businesses, 62.5% (n=243) were inspected by public health authorities, 33.1% (n=129) by the EFET, and 3.3% (n=13) by another governmental authority. In 52.3% (n=273) of the total sample, non-compliances related to general hygiene were reported; in 48.7% (n=254), HACCP procedures were implemented; and in 1% (n=5), unsafe food was detected.

## Results

Regarding the participants’ demographic characteristics, 70.1% (n=366) were male, and 93.1% (n=486) were Greek. The data showed that 35.1% (n=183) of individuals were between the ages of 40 and 49, while 32.8% (n=171) were between the ages of 30 and 39. More than half (59.4%, n=310) were high school or vocational training graduates, 26.6% (n=139) were university/technological institute graduates, and 7.5% (n=39) held a master’s or PhD degree. Of the participants, 38.9% (n=203) were business owners, while 30.8% (n=161) held positions as department supervisors or managers. A total of 64.8% (n=338) had received training in food hygiene, with 40.6% (n=212) having official training in programs approved by EFET and 17.8% (n=93) being trained in their workplace. With respect to work experience, 25.5% (n=133) had been working at their current company for 6-10 years, and 24.9% (n=130) for 11-15 years. Regarding business self-auditing, 67.2% (n=351) met the prerequisite requirements, 17.4% (n=91) followed a company-specific HACCP plan, and 10.3% (n=54) followed a generic HACCP plan. Demographic and occupational characteristics are presented in Table 1.

**Table 1:** Sample characteristics * Calculated based on those who had been assessed. GHPs: good hygiene practices; HACCP: Hazard Analysis and Critical Control Points; CTF: Common Training Framework

Characteristics		N	%
Type of catering establishment	Full meal service	300	57.5
	Snack (cafés, bars, refreshment bars, etc.)	222	42.5
Geographical area	Eastern sector of Attica	78	14.9
	Northern sector of Athens	103	19.7
	Western sector of Attica	76	14.6
	Central sector of Athens	102	19.5
	Southern sector of Athens	80	15.3
	Piraeus & Islands	83	15.9
Maximum number of customers, mean (SD), median (IQR)		221.7 (336.7)	120.0 (50-250)
Number of employees	1-50	474	90.8
	51-250	30	5.7
	251 or more	18	3.4
Audits carried out by a public audit service (last five years)	No	132	25.3
	Yes	390	74.7
Control service*			
Hellenic Food Authority		173	33.1
Public Health Authority		326	62.5
Other public service		17	3.3
Type of non-compliances*			
No non-compliances observed		98	18.8
General hygiene (GHPs)		273	52.3
HACCP procedures		254	48.7
Unsafe food		5	1.0
Gender	Male	366	70.1
	Female	156	29.9
Age	18-39	209	40.0
	40-49	183	35.1
	50	130	24.9
Nationality	Greek	486	93.1
	Other	36	6.9
Educational level	Primary/Secondary School	34	6.5
	High School-Vocational Training Institute	310	59.4
	University/Technical university	139	26.6
	Master/PhD	39	7.5
Position in the company	Management executive staff	85	16.3
	Head chef	49	9.4
	Business owner	203	38.9
	Department supervisor	161	30.8
	Other positions	24	4.6
Have you been trained in food hygiene?	No	184	35.2
	Yes	338	64.8
What education/training have you received in food hygiene?			
Formal training course - Hellenic Food Authority CTF		212	40.6
Workplace training		93	17.8
Training Course		40	7.7
Educational competence through studies		60	11.5
Other		8	1.5
Years of experience in the company	Up to 5	98	18.8
	6-10	133	25.5
	11-15	130	24.9
	16	161	30.8
Type of control activity based on HACCP principles that your company follows	Guide to Good Practices/Generic HACCP Plan	54	10.3
	Don’t know	26	5.0
	Prerequisite Requirements (Cleaning, Pest Control/Rodent Control)	351	67.2
	Company-Specific HACCP Plan	91	17.4
In what type of food establishment have you worked in the past?	Bakery/pastry	47	9.0
	Mass catering/food service	479	91.8
	Food industry	21	4.0
	Small-scale food production	23	4.4
	Retail trade	35	6.7
	Primary production	6	1.1
	No previous experience	17	3.3

Knowledge and attitude assessment in food hygiene

**Figure 1:**
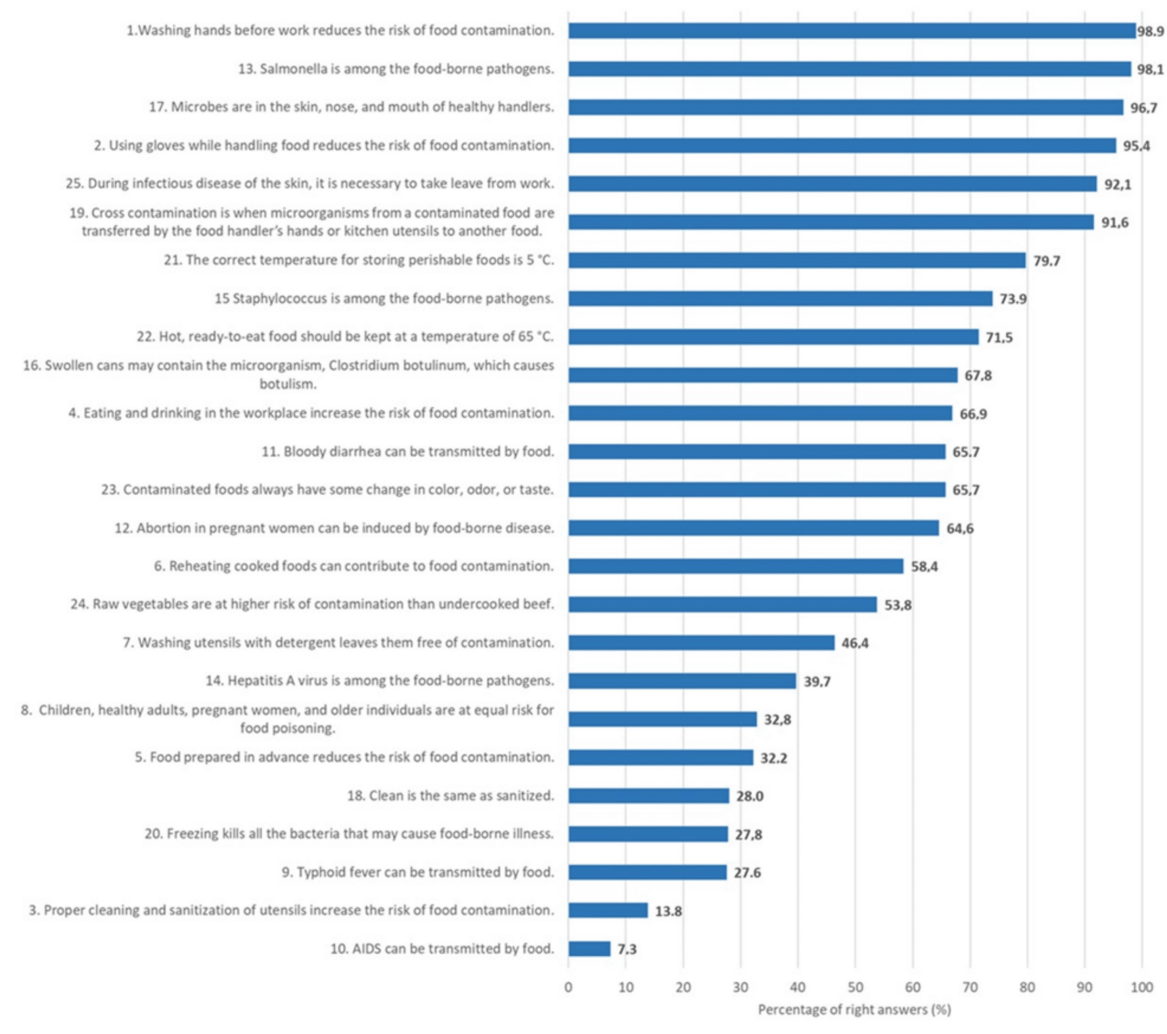
Percentages of responses to food hygiene knowledge statements, presented in descending order

**Figure 2:**
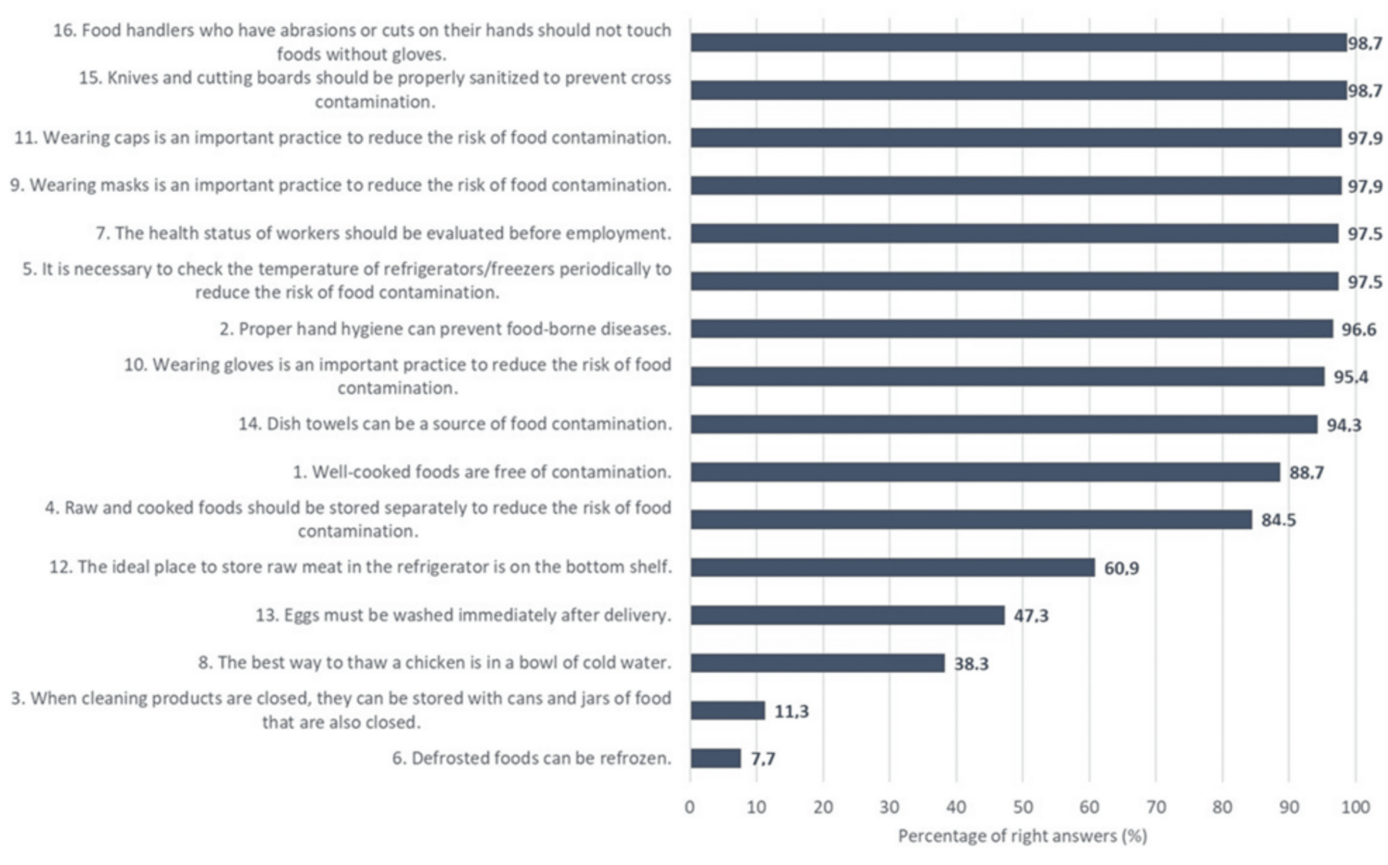
Percentages of responses to food hygiene attitude statements, presented in descending order

Participants’ answers on the knowledge and attitudes questionnaires regarding personal hygiene, cross-contamination, foodborne diseases carried by microor­ganisms, temperature control, and hygiene practices are presented in Figures 1-2.

The highest percentage of correct answers, 98.9% (n=516), was observed in relation to washing hands before handling food. This was followed by correct answers regarding *Salmonella* as a foodborne pathogen (98.1%, n=512) and that bacteria can be found on the skin, hair, nose, and mouth of healthy workers (96.7%, n=505). In addition, 95.4% (n=498) correctly answered that wearing gloves when handling food reduces the risk of contamination. On the other hand, fewer correct answers were given regarding the following: contaminated food always shows changes in color, smell, or taste (32%, n=167); a vegetable salad is more microbiologically dangerous than undercooked beef (31%, n=162); and typhoid fever can be transmitted through food (27.6%, n=144).

Regarding the attitude questionnaire, almost all participants agreed that knives and cutting boards should be properly disinfected to prevent cross-contamination and that people with cuts or scratches on their hands should not touch food without gloves (98.7%, n=515 in both cases). In addition, 97.9% (n=511) correctly agreed that wearing gloves is an important practice for reducing the risk of food contamination and that wearing protective clothing is a good practice for reducing the risk of food contamination. Fewer correct answers were given regarding the ideal place to store raw meat in the refrigerator, which is on the lowest shelf (60.9%, n=318); the best way to defrost a chicken, which is in a bowl of cold water (45.6%, n=238); and that eggs should be washed immediately after purchase (32.8%, n=171).

**Figure 3:**
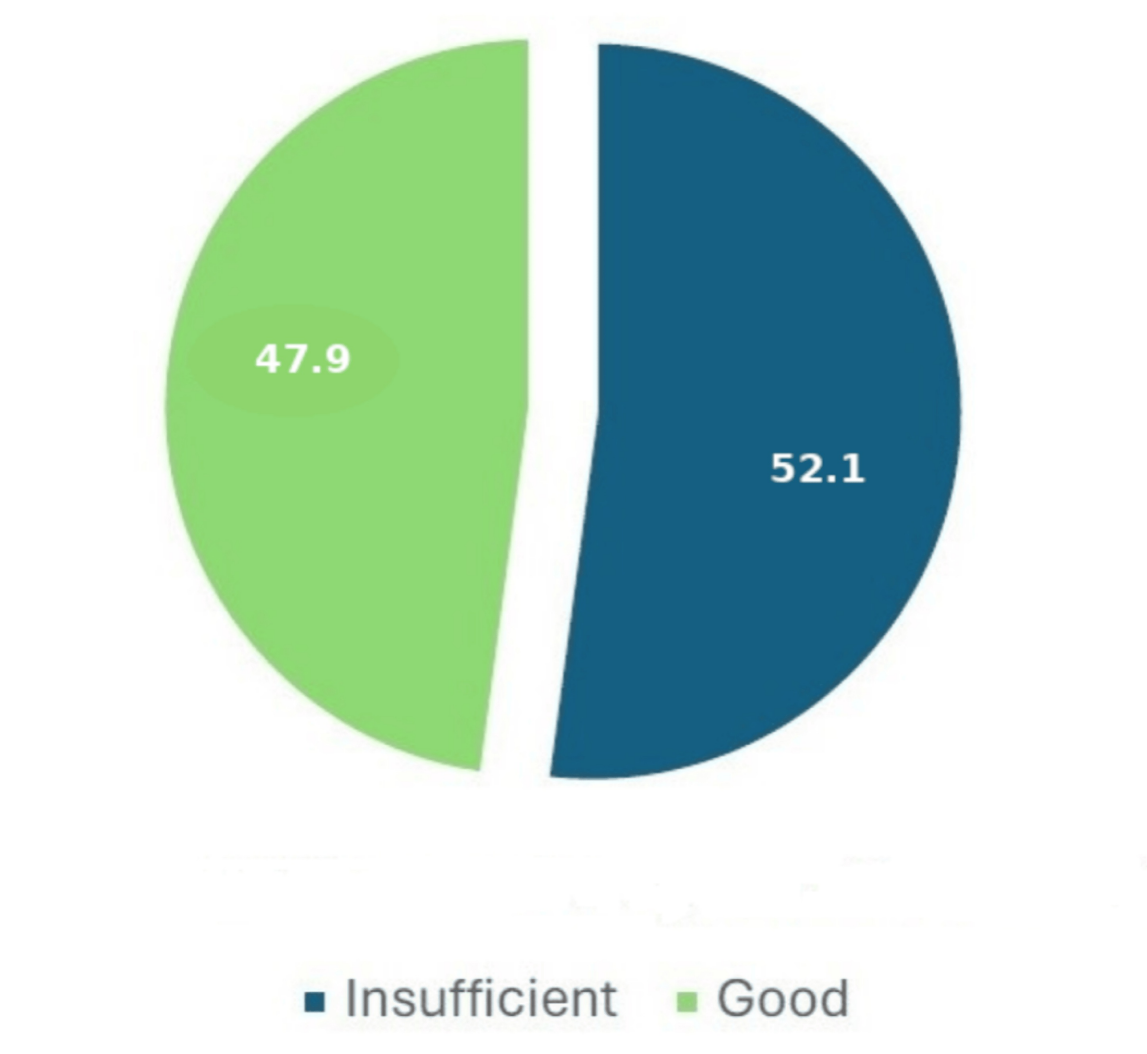
Distribution of food hygiene knowledge levels (%)

**Figure 4:**
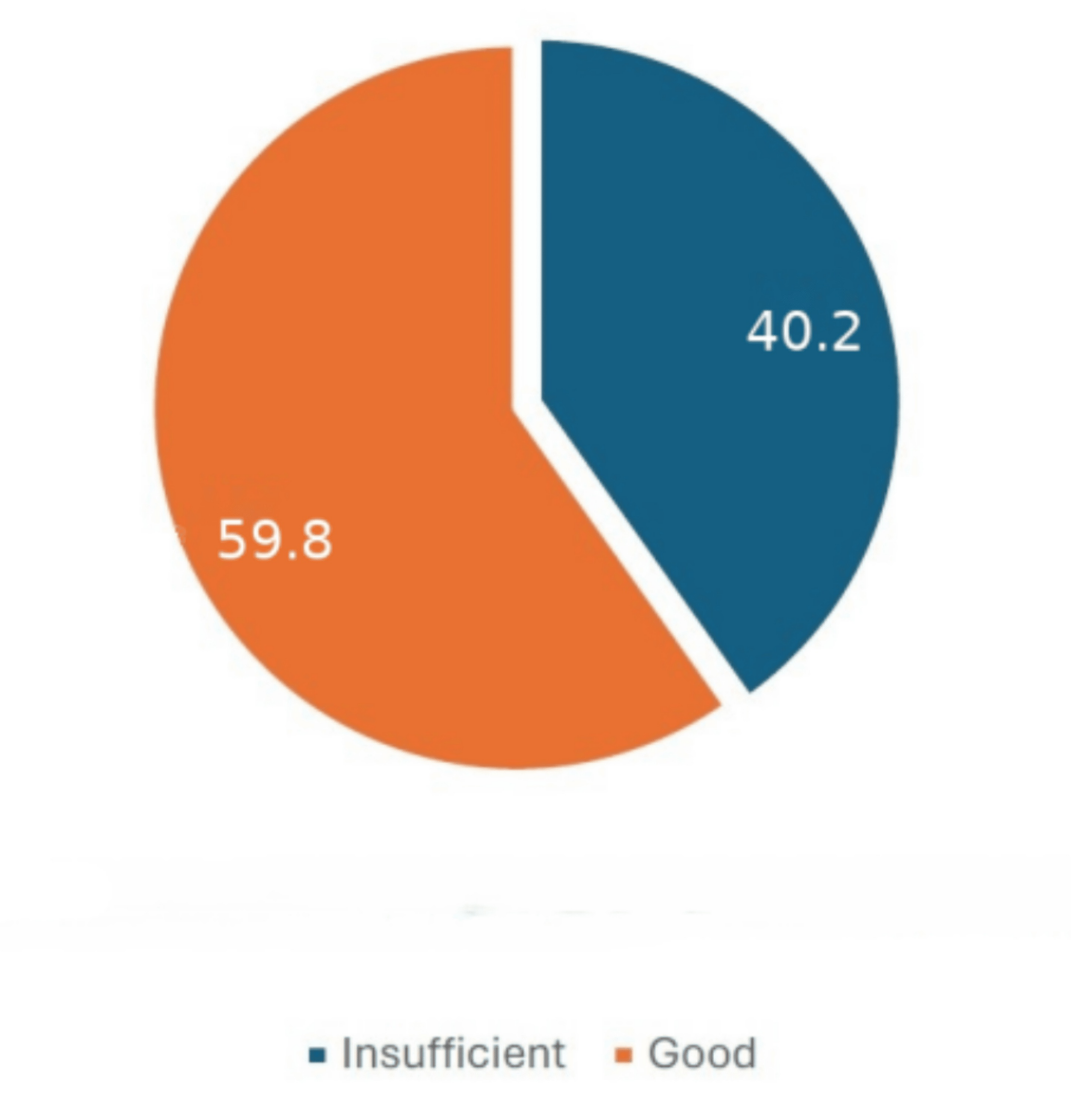
Distribution of food hygiene attitude levels (%)

Knowledge was assessed on a scale ranging from 0 to 25 points. A total of 47.9% (n=250) had scores equal to or greater than 18 points and were considered to have good knowledge (Figure 3). Similarly, attitude scores ranged from 0 to 16 points, and participants who answered 12 or more questions correctly were considered to have a good understanding. In this sample, 94.3% (n=492) had a good understanding of food hygiene. Due to the small number of participants with scores below 12 on the attitude scale, it was decided to divide the participants based on the median score of 14 points. Based on this cutoff (≥14), 59.8% (n=312) were considered to have a good understanding of food hygiene and safety (Figure 4).

The number of employees, age, educational level, role in the company, and type of control were associated with the level of knowledge concerning food hygiene (Table 2). Employees in companies with 51 or more people on the working staff were 2.68 times more likely to have good knowledge of food hygiene than those working in companies with fewer staff (OR=2.68, 95% CI: 1.17-6.13). Odds of good knowledge were also higher for participants 40-49 years old in comparison with those being 18-39 (OR=1.94, 95% CI: 1.24-3.01). For those having a master’s degree or PhD, it was 5.04 times more possible to have good knowledge on food hygiene than for those being a graduate of primary or secondary school (OR=5.04, 95% CI: 1.58-16.06). The owners and the executive managers were 64% and 62%, respectively, less likely to have a good level of knowledge about food hygiene than head chefs (OR=0.36, 95% CI: 0.15-0.86; OR=0.38, 95% CI: 0.17-0.85). Employees who were unaware of the control measures used in their workplace and those working in businesses that met only the minimum required specifications were 85% and 67% less likely, respectively, to have a good level of food hygiene knowledge compared to employees in businesses with a control system based on a good practice guide/generic HACCP plan (OR=0.15, 95% CI: 0.05-0.49; OR=0.33, 95% CI: 0.16-0.68).

**Table 2:** Results of logistic regression analyses for food hygiene knowledge ^+^ Odds ratio (95% confidence interval); Bold values indicate statistical significance (p < 0.05). HACCP: Hazard Analysis and Critical Control Points

	OR (95% CI)^+^	P
Type of catering establishment (snack vs. full meal)	1.13 (0.74-1.75)	0.567
Number of employees (51 or more vs. 1-50)	2.68 (1.17-6.13)	0.020
Audits carried out by a State Control Service (Yes vs. No)	1.15 (0.72-1.83)	0.566
Gender (Female vs. Male)	0.76 (0.5-1.16)	0.200
Age: 40-49 vs. 18-39	1.94 (1.24-3.01)	0.003
Age: 50+ vs. 18-39	1.26 (0.76-2.1)	0.375
Nationality (Other vs. Greek)	0.52 (0.23-1.17)	0.114
Educational level: High School-Vocational Training Institute vs. Primary/Secondary School	1.46 (0.61-3.47)	0.392
Educational level: University/Technical university vs. Primary/Secondary School	2.29 (0.92-5.69)	0.074
Educational level: Master/PhD vs. Secondary School	5.04 (1.58-16.06)	0.006
Position Management: Executive Staff vs. Head Chef	0.36 (0.15-0.86)	0.021
Position: Business Owner vs. Head Chef	0.38 (0.17-0.85)	0.018
Position: Department Supervisor vs. Head Chef	0.58 (0.26-1.27)	0.173
Position: Other vs. Head Chef	0.33 (0.10-1.09)	0.069
Have you been trained in food hygiene? (Yes vs. No)	1.34 (0.86-2.07)	0.195
Type of self-control activity: Don’t know vs. Guide to Good Practices/Generic HACCP Plan	0.15 (0.05-0.49)	0.002
Type of control activity: Prerequisite Requirements (Cleaning, Pest Control/Rodent Control) vs. Guide to Good Practices/Generic HACCP Plan	0.33 (0.16-0.68)	0.003
Type of control activity: Company-Specific HACCP Plan vs. Prerequisite Requirements (Cleaning, Pest Control/Rodent Control)	0.59 (0.26-1.34)	0.204
Bakery/Pastry (Yes vs. No)	0.75 (0.32-1.74)	0.504
Mass Catering/Food Service (Yes vs. No)	1.34 (0.62-2.92)	0.457
Food Industry (Yes vs. No)	2.45 (0.78-7.66)	0.124
Small-Scale Food Production (Yes vs. No)	0.96 (0.33-2.76)	0.937
Retail Trade (Yes vs. No)	1.05 (0.43-2.57)	0.919

In this sample, 94.3% (n=492) had a good level of attitudes toward food hygiene. Due to the small number of participants with scores below 12 on the attitude scale, it was decided to divide the participants based on a median value of 14 points. Based on this cutoff (≥14), 59.8% (n=312) were considered to have a good understanding of food hygiene (Figure 2).

Logistic regression analysis

Multiple logistic regression analysis was conducted, and after adjusting demographic and occupational characteristics for participants, it was found that the number of employees, age, educational level, position in the company, and the type of control were associated with the level of knowledge concerning food hygiene (Table 2). Employees in companies with 51 or more people on the working staff were 2.68 times more likely to have good knowledge of food hygiene than those working in companies with fewer staff (OR=2.68, 95% CI: 1.17-6.13). Odds of good knowledge were also higher for participants 40-49 years old in comparison with those being 18-39 (OR=1.94, 95% CI: 1.24-3.01). For those having a master’s degree or PhD, it was 5.04 times more possible to have good knowledge on food hygiene than for those being a graduate of primary or secondary school (OR=5.04, 95% CI: 1.58-16.06). The owners and the executive managers were 64% and 62%, respectively, less likely to have a good level of knowledge about food hygiene than head chefs (OR=0.36, 95% CI: 0.15-0.86; OR=0.38, 95% CI: 0.17-0.85). Employees who were unaware of the control measures used in their workplace and those working in businesses that met only the minimum required specifications were 85% and 67% less likely, respectively, to have a good level of food hygiene knowledge compared to employees in businesses with a control system based on a good practice guide/generic HACCP plan chefs (OR=0.15, 95% CI: 0.05-0.49; OR=0.33, 95% CI: 0.16-0.68).

Results were similar for the level of correct attitudes towards food hygiene (Table 3). Participants working in businesses with 51 or more employees were 3.01 times more likely to have a good level of food hygiene attitudes compared to employees in businesses with fewer staff (OR=3.01, 95% CI: 1.11-8.19). Employees in services that had been inspected in the last five years were 1.75 times more likely to have correct food hygiene attitudes compared to those working in services that had not been inspected (OR=1.75, 95% CI: 1.08-2.81). Education was also a significant predictor. Graduates of Master's/PhD programs were 5.16 times more likely to have a strong understanding compared to those who graduated from primary/secondary school (OR=5.16, 95% CI: 1.49-17.92). Managers, business owners, and supervisors were 82%, 69%, and 78% less likely, respectively, to have an adequate level of attitudes about food hygiene compared to employees who were head chefs (OR=0.18, 95% CI: 0.07-0.43; OR=0.31, 95% CI: 0.14-0.68; OR=0.22, 95% CI: 0.10-0.49). Finally, employees with previous experience in the food industry were 12.96 times more likely to exhibit a high level of awareness and a positive outlook on food hygiene compared to those without such experience (OR=12.96, 95% CI: 1.54-109.09).

**Table 3:** Results of logistic regression analyses for food hygiene attitudes + Odds ratio (95% confidence interval); Bold values indicate statistical significance (p < 0.05). HACCP: Hazard Analysis and Critical Control Points

	OR (95% CI)^+^	P
Type of catering establishment (snack vs. full meal)	1.44 (0.92-2.24)	0.110
Number of employees (51 or more vs. 1-50)	3.01 (1.11-8.19)	0.030
Audits carried out by an official control service (Yes vs. No)	1.75 (1.08-2.81)	0.022
Gender (Female vs. Male)	0.81 (0.53-1.25)	0.342
Age: 40-49 vs. 18-39	1.2 (0.76-1.9)	0.426
Age:50+ vs. 18-39	1.61 (0.95-2.75)	0.079
Nationality (Other vs. Greek)	0.87 (0.39-1.91)	0.725
Educational level: High School-Vocational Training Institute vs. Primary/Secondary School	1.53 (0.65-3.58)	0.330
Educational level: University/Technical university vs. Primary/Secondary School	1.65 (0.67-4.05)	0.275
Educational level: Master/PhD vs. Secondary School	5.16 (1.49-17.92)	0.010
Position Management executive staff vs. Head Chef	0.18 (0.07-0.43)	<0.001
Position: Business Owner vs. Head Chef	0.31 (0.14-0.68)	0.004
Position: Department Supervisor vs. Head Chef	0.22 (0.1-0.49)	<0.001
Position: Other vs. Head Chef	2.04 (0.45-9.28)	0.357
Have you been trained in food hygiene? (Yes vs. No)	1.83 (1.17-2.86)	0.008
Type of control activity: Don’t know vs Guide to Good Practices/Generic HACCP Plan	0.86 (0.27-2.79)	0.802
Type of control activity: Prerequisite Requirements (Cleaning, Pest Control/Rodent Control) vs. Guide to Good Practices/Generic HACCP Plan	1.05 (0.5-2.18)	0.905
Type of control activity: Company-Specific HACCP Plan vs. Prerequisite Requirements (Cleaning, Pest Control/Rodent Control)	2.17 (0.9-5.22)	0.083
Bakery/Pastry (Yes vs. No)	1.14 (0.46-2.79)	0.777
Mass Catering/Food Service (Yes vs. No)	1.34 (0.61-2.95)	0.471
Food Industry (Yes vs. No)	12.96 (1.54-109.09)	0.018
Small-Scale Food Production (Yes vs. No)	0.47 (0.15-1.48)	0.194
Retail Trade (Yes vs. No)	0.48 (0.19-1.24)	0.131

## Discussion

The findings of our research highlight several critical factors that influence the knowledge and attitudes of FBOs in the Attica region on food hygiene.

Factors influencing knowledge of food hygiene

The demographic profile of participants, which is dominated by men (70.1%) and people aged 30-49, probably reflects the traditional professional hierarchy in the Greek catering sector, where decision-making roles, such as business owners and managers, who make up the majority of our sample, are mainly held by men. Similar gender-based data on managerial and head chef positions have been recorded in surveys conducted in Brazil and Portugal [25,56].

The significant difference in knowledge levels observed between FBOs in medium-sized and large enterprises (p=0.020) suggests that business size is an indicator of organizational maturity. Larger businesses typically have the financial and administrative capacity to implement more systematic employee training and strict self-monitoring procedures based on HACCP principles, which are often less standardized in smaller facilities [45,46,57,58]. Similarly, the finding that the 40-49 age group showed a higher knowledge level (p=0.003) indicates that professional maturity and years of cumulative exposure to a variety of operational challenges contribute directly to the effective enforcement and implementation of food safety standards [45,46,57].

The crucial role of educational level further highlights the complexity of knowledge acquisition. The significant advantage of postgraduate or doctoral degree holders in relation to high school graduates (p=0.006) confirms that higher academic education provides a stronger knowledge base [45,46,57,59]. This significant gap highlights the critical need for targeted, simplified training for personnel with a lower educational background to ensure consistent compliance with safety protocols [38].

The crucial role of ethnicity and educational level further highlights the complexity of knowledge attainment. Lower levels of knowledge among non-Greek FBOs highlight potential cultural or lingual barriers in existing training programs [60]. This highlights the need for culturally adapted training modules to be beneficial to those who may not have extensive knowledge due to their ethnic background [61].

The professional role within the business emerged as a key factor influencing expertise. Personnel in roles of managers and head chefs demonstrated significantly better knowledge (p=0.021 and p=0.018, respectively), a result mainly attributed to their direct responsibility for supervising food safety. This knowledge ladder ensures that those responsible for operational oversight have the expertise necessary to maintain GHPs [45,46,57].

The systematic application of HACCP principles, along with GHPs guidelines, is a key factor in the effectiveness of businesses and has a big impact on the professional performance of FBOs. Our findings indicate clearly that the type of self-control system implemented is a strong factor in predicting knowledge levels. Specifically, the significantly lower knowledge observed among participants who were unaware of their business's HACCP status (p=0.002) indicates a significant disconnect between the management protocols and employees' awareness.

Surveys show that respondents who are unsure about HACCP implementation often lack the basic knowledge required to effectively apply GHP. This lack of clarity likely stems from inadequate training or a lack of understanding of internal food safety protocols, which can lead to a failure to identify critical control points (CCPs) during daily operations [57]. This lack of clarity often turns a safety system into a bureaucratic burden rather than a functional tool for hazard prevention. Therefore, the relationship between uncertainty about HACCP and lower levels of knowledge underscores the vital importance of effective food hygiene training as a catalyst for behavioral change and food safety [59].

Official control was associated with participants’ knowledge of food hygiene. Specifically, inspections conducted by public health authorities were associated with significantly higher knowledge levels (p=0.019), whereas official inspections overall were not. Inspections can be perceived by FBOs as beneficial, fostering a better understanding of regulations and hazards related to food safety [62,63]. This association suggests that the role of the inspector is evolving from a strictly punitive figure to a training partner who clarifies complex legal requirements. Rizzo et al. (2025) emphasized the role of inspections in motivating food businesses to adopt comprehensive food safety management strategies, enhancing hygiene standards and the overall food safety culture [64]. This interaction between food businesses and public health authorities can create a dynamic environment that promotes a better understanding of compliance requirements [65], effectively transforming a passive inspection into a continuous learning process that reduces operational uncertainty. Furthermore, evidence suggests that the transparency created by regularly publishing inspection results encourages businesses to maintain higher standards, thereby reducing foodborne risk [66,67]. This public reporting system acts as a powerful motivation for FBOs to integrate food safety as a core value of their business model rather than simply as a regulatory requirement.

Among participants with a satisfactory level of knowledge, the largest percentage had received training in food hygiene through formal training organized by the EFET. These percentages suggest that formal training is the main pillar of knowledge, but the presence of a significant percentage of trained individuals with "satisfactory" rather than "excellent" knowledge highlights the need for repeated and more interactive training courses. According to Cavalli and Salay (2007), the level of formal training, participation in training programs, and positive professional experience all contribute to food safety [68]. The combination of academic background and specialized training creates a strong defense mechanism against foodborne hazards. Brown et al. (2014) examined food safety knowledge among restaurant staff and reported that managers who had received food safety training demonstrated better knowledge, indicating a link between formal training and better knowledge retention [69]. This link suggests that training is most effective when it is role-specific. For managers, it enhances their supervisory ability, while for operational staff, it translates into safer handling practices. Without continuous reinforcement, even formally trained personnel can experience knowledge decay, which directly threatens the food safety culture of the business.

Personal hygiene is vital for preventing food contamination and foodborne illnesses, as inadequate hand washing is responsible for more than 25% of food contamination [70-72]. The results of this study show that participants have a high level of knowledge about personal hygiene, especially hand hygiene and wearing gloves when handling food. A study in Addis Ababa reported that hand washing and glove use were recognized by the vast majority of participants [73]. Similarly, Akabanda et al. (2017) reported that 77.9% of food handlers in institutions recognized the importance of wearing gloves, while a very high percentage (98.7%) reported good knowledge of basic GHPs such as hand washing [60]. This high level of theory-based awareness suggests that basic hygiene protocols are well known, but consistent implementation remains the main challenge for public health.

Findings on cleaning and disinfection indicate sufficient knowledge of these topics and their crucial role in equipment disinfection; however, a knowledge gap has been identified regarding the effect of detergents on the removal of microorganisms from equipment, an important issue for the implementation of GHP. A study in Nigeria highlighted that, while street food vendors recognized the importance of hygiene, a significant percentage did not understand how detergents work effectively against microorganisms [74]. Another study revealed that the mechanism by which detergents reduce microbial burdens on surfaces is often overlooked, resulting in inadequate cleaning practices [75]. This lack of mechanical and chemical understanding is a critical finding, as evidence suggests that mechanical cleaning is required prior to disinfection to ensure effectiveness [76].

Regarding knowledge of food handling, participants showed moderate awareness of the relationship between preparing meals in advance and increased food safety risks. Specifically, when food is prepared in advance and not stored properly, there is a risk of microbial growth due to inappropriate temperatures and time delays, which can lead to the formation of harmful toxins [77,78]. Evidence confirms that improper storage conditions are significantly associated with microbial growth in ready-to-eat foods, making strict temperature control essential [78,79]. In addition, participants demonstrated insufficient knowledge regarding the incorrect assumption that contaminated foods usually show visible changes in color, odor, or taste. Studies have shown that foodborne pathogens can be present without noticeable changes, highlighting the need for surveillance practices beyond visual inspection [80,81]. Even foods that maintain their organoleptic characteristics can host pathogens, making it essential to provide systematic training in food safety [82,83].

Cross-contamination - the transfer of bacteria between foods, from hands to food, or from equipment to food - is one of the most common causes of outbreaks [84]. Participants were generally familiar with the concept, but demonstrated only moderate knowledge about the increased risk of contamination from consuming food and beverages in the workplace. In the study by Alqurashi et al. (2019), food service personnel demonstrated high knowledge of cross-contamination, with 77.9% fully understanding the concept [85]. However, Siddiky et al. (2024) identified cross-contamination as a continuing challenge in food service environments, where FBOs often fail to implement the necessary preventive measures despite their theoretical knowledge, emphasizing that inadequate training does not translate into improved HACCP [86].

In view of the knowledge about pathogens and foodborne diseases, FBOs have demonstrated significant awareness of *Salmonella*, an important cause of non-typhoidal salmonellosis (NTS) transmitted through food. NTS infections are responsible for approximately 93.8 million cases worldwide annually and are mainly associated with the consumption of poultry and eggs [87]. However, knowledge is moderate to insufficient for several highly important foodborne pathogens, including hepatitis A virus (HAV), *S. aureus*, *Escherichia coli *O157, *Clostridium botulinum*, *Salmonella typhi*, and the potential implications of *L. monocytogenes* infection during pregnancy, confirming significant knowledge gaps and inadequate training of personnel.

*L. monocytogenes* is a well-known pathogen that causes listeriosis, a serious foodborne illness with high mortality, especially in vulnerable populations such as pregnant women [88]. Consumption of contaminated food can lead to serious complications during pregnancy [71,89,90,91]. Studies show that listeriosis outbreaks can affect a significant proportion of pregnant women infected with *L. monocytogenes*, leading to spontaneous fetal loss or severe neonatal infection [71,91]. Our findings underscore that FBOs demonstrated only a moderate level of knowledge about *L. monocytogenes* and its implications and often did not recognize it as a serious threat to public health.

A notable gap was observed with regard to foodborne botulism. Participants reported only moderate knowledge about the possible presence of dangerous neurotoxins in swollen cans. *C. botulinum* causes a very serious and potentially fatal disease characterized by neuroparalysis due to the presence of botulinum neurotoxins (BoNTs) [92,93]. Published statistics show that restaurants, delicatessens, and cafeterias account for a significant proportion of reported cases [94]. The frequency of the disease highlights the need for improved GHP in food establishments [94].

Shiga toxin-producing *E. coli* (STEC) strains are pathogenic strains associated with clinical diseases ranging from mild diarrhea to bloody diarrhea (BD) and hemolytic uremic syndrome (HUS) [95]. *E. coli* O157:H7 remains an important foodborne pathogen worldwide and is mainly associated with the consumption of beef [48]. The relevant question revealed knowledge gaps regarding *E. coli* O157:H7 and other dangerous microorganisms (e.g., *Trichinella spiralis*): a significant proportion of FBOs consider vegetable salads to be more microbiologically hazardous, even though EU microbiological criteria make ready-to-eat salads safe when compared to undercooked beef [96]. Nevertheless, *E. coli* O157:H7 has been isolated from leafy vegetables, highlighting that both animal products and vegetables can act as transmission vectors [97-99].

Knowledge about hepatitis A as a foodborne pathogen was also insufficient. Similar findings were reported by Onyeneho and Hedberg (2013), who reported that only 21% of catering workers in restaurants were aware of hepatitis A. Similarly, Ansari-Lari et al. (2010) reported that employees often did not know whether HAV and* Staphylococcus* are foodborne, indicating a significant knowledge gap [47,100]. Similarly, an inadequate level of knowledge has been reported for *S. typhi*, the causative agent of typhoid fever, which is mainly transmitted through contaminated water and food [101,102]. Global estimates suggest 11-26 million cases per year [103]. Limited surveillance by public health authorities and inadequate personnel training exacerbate these knowledge gaps in food hygiene [104].

Factors influencing attitudes toward food hygiene

FBOs' attitudes toward food safety appear to be significantly affected by specific demographic and professional factors, as indicated by our study findings. FBOs working in businesses with more than 50 employees (medium and large) demonstrated significantly more positive attitudes than those working in small businesses (p=0.030). This difference suggests that larger organizational structures can encourage a stronger food safety culture through stricter internal control procedures and dedicated resources, which are often lacking in smaller establishments. The implication is that the resource constraints associated with size in small businesses may lead to a less hierarchical approach to hygiene protocols.

Supporting this view, other studies have shown that small businesses are more prone to non-compliance with hygiene standards than larger businesses, suggesting that smaller size increases the likelihood of non-compliance [105]. Similarly, in Qatar, larger businesses demonstrated better adherence to food safety procedures. Specifically, restaurants with "occasional sit-down dining" had better record-keeping for CCPs than fast food restaurants [106]. This suggests that the complexity and scale of a business require more formal and positive attitudes towards safety management systems. Furthermore, another study on attitudes toward official food control reported that small FBOs rated official control lower than larger FBOs [22].

On the contrary, it is important to note that size does not always guarantee superior compliance. A study of restaurants in Attica with fully trained personnel, including food safety supervisors, revealed that small businesses demonstrated greater compliance than medium and large businesses [50]. This discrepancy may occur because large-scale catering involves significantly more complex food storage and handling operations than fast-food outlets, increasing the likelihood of operational errors despite a positive theoretical attitude [107]. This highlights that while larger businesses may have better "attitudes," the practical implementation of GHPs may be hampered by the overwhelming complexity of their processes.

The frequency and perception of official inspections significantly shape FBO attitudes toward compliance with food safety regulations. In the present study, participants whose businesses were inspected by public health authorities more than once a year showed significantly more positive attitudes toward food hygiene compared to those who were inspected less frequently or not at all (p=0.005). This suggests that regular government inspections serve as a constant reminder of regulatory obligations, reinforcing a preventive attitude. In addition, a positive correlation was observed between FBO attitudes and their levels of knowledge, reinforcing the idea that a well-informed operator is more likely to appreciate and implement safety protocols.

This finding is consistent with previous research in Attica, which noted that business owners perceive consistent inspection programs as a support mechanism rather than a punishing process [50]. Official inspections can be considered beneficial as they provide a structured approach to identifying hazards that may be overlooked during routine operations [62]. However, the impact of these inspections is often mediated by the inspection climate. For example, when FBOs perceive inspectors as training partners, their willingness to adopt higher safety standards increases significantly [108]. Conversely, a lack of regular inspections can lead to a decline in the perceived importance of GHPs, where safety measures are seen as optional rather than necessary [109]. Therefore, the frequency of inspections is not merely a regulatory measure, but a critical factor in maintaining a positive food safety culture in the catering sector.

Higher education was strongly associated with more positive attitudes: participants with a master's/doctorate degree were significantly more likely to have positive attitudes than those with secondary education (p=0.010). This finding supports the hypothesis that a higher education level enhances awareness of the importance of food hygiene. Beyond acquiring technical knowledge, advanced education likely encourages critical thinking and a deeper understanding of the long-term effects of foodborne hazards on public health. This knowledge base makes highly educated FBOs more receptive to safety protocols, as they perceive them not as restrictive rules but as basic scientific requirements for consumer health protection.

Similarly, Chen et al. (2018) reported that higher education is associated with positive attitudes among dairy factory workers [48]. Α higher level of education is positively associated with employees' attitudes [110,111]. This association suggests that individuals with higher educational backgrounds may be more receptive to the scientific rationale behind safety protocols, making them more likely to internalize these practices as a professional duty rather than a mandatory task. This aligns with findings from Malaysia showing that educational attainment directly influences attitudes toward food hygiene and food safety practices among food handlers [111]. This finding suggests that for staff with lower levels of education, training programs need to be specifically tailored to bridge this gap, ensuring that the importance of food safety is effectively communicated regardless of initial academic background.

Professional roles in a food business were also significantly associated with attitudes toward food hygiene. In our study, managers and head chefs demonstrated more positive attitudes compared to other employee members (p=0.021 and p=0.018, respectively). This variation likely stems from the higher level of accountability and legal responsibility that is inherent in leadership roles, which requires these professionals to internalize food safety as a core business value rather than a simple set of rules. Such positive attitudes are critical, as leaders serve as role models, directly influencing the food safety culture of the entire establishment [45,46,57].

Furthermore, research has repeatedly shown that those in supervisory positions are more likely to prioritize safety protocols because they are more aware of the serious legal and financial consequences of a foodborne outbreak. Management commitment is the keystone of an effective food safety management system. Without a positive attitude at the highest level, the implementation of GHPs at the operational level is often compromised [112]. Administrative-level behaviours may not translate into on-the-ground practices [113,114]. This means that while the positive attitudes of managers in Attica are encouraging, the challenge remains to bridge the gap and to cultivate a similarly strong attitude among employees who are involved in the direct handling of food but may not feel the same degree of professional responsibility [45,46,57].

Our study confirms the critical role of education in shaping positive attitudes toward food hygiene. Food hygiene training had a positive effect on attitudes: FBOs who attended training programs had more positive attitudes than those who did not attend (p=0.008). This finding suggests that training acts as a catalyst for the transition from simple compliance to internal commitment, reinforcing the perception that hygiene is an integral part of professional ethics. This highlights training as a key factor in strengthening internal commitment and building a strong food safety culture in the mass catering service.

Hassan and Fweja (2020) emphasized the importance of training programs for improving knowledge and attitudes among food business personnel in Malaysia [115] and reported statistically significant improvements in attitudes among trained personnel. The consequence of these improvements is that structured training reduces the psychological distance between operators and safety protocols, making them more proactive in preventing risks. Similarly, Bailey et al. (2015) reported that regular training sessions were instrumental in shaping positive attitudes toward food allergy safety among food handlers in restaurants [116]. Therefore, continuous training is not just a regulatory obligation but a strategic tool that cultivates a mindset of constant awareness, which is essential for reducing food-borne hazards in mass catering environments.

Professional experience in the food industry was associated with significantly more positive attitudes (p=0.018), highlighting the role of professional expertise and exposure to high-demand safety environments. This suggests that long-term experience with businesses allows FBOs to develop a deeper understanding of risks, transforming formal compliance into a conscious professional attitude. Mulat et al. (2024) reported that training combined with work experience positively influences GHP, indicating a clear relationship between experience and improved attitudes toward hygiene [117].

The cumulative effect of years of experience in the field acts as a form of practical wisdom that complements theoretical education. Lee and Seo (2020) examined the impact of professional experience on food safety and hygiene management practices and concluded that employee experience is critical to shaping attitudes and implementing GHPs [46]. The implication of this finding is that experience acts as a protective factor for public health, as experienced professionals are more likely to anticipate and prevent failures in the food chain. Therefore, encouraging the retention of experienced staff in food service businesses can be essential to the stability and improvement of food safety.

Study limitations

This study provides valuable insights into the food safety landscape in Attica, but certain limitations must be acknowledged to ensure a balanced interpretation of the findings. First, the cross-sectional design of the study limits our ability to extract causal conclusions about the relationships between demographic factors, knowledge, and attitudes. Furthermore, as the study was based on self-reported data, the results may be subject to social desirability bias, whereby participants may report more positive attitudes or higher compliance than is actually the case.

Furthermore, as the study focused on FBOs rather than food handlers, the findings reflect the perspective and theoretical awareness of management rather than actual operational practices at the food preparation level. Finally, while Attica is the largest urban area in Greece, the findings may not be fully generalizable to rural areas or different cultural contexts within the country. Despite these limitations, the use of a representative sample and validated statistical analyses provides a solid basis for understanding the administrative and leadership factors that shape food safety culture.

## Conclusions

This study provides a comprehensive assessment of the food safety environment in Attica, offering critical insights into the knowledge and attitudes of FBOs. Our findings indicate that, while there is a strong knowledge base regarding basic hygiene (such as hand washing and awareness of *Salmonella*), there are still significant gaps in knowledge regarding complex microbiological hazards, including *L. monocytogenes*, *C. botulinum*, and specific chemical disinfection mechanisms. From a public health perspective, this study contributes to the field by mapping the educational and professional factors that shape food safety culture at the management level. Recognizing that company size, higher educational level (master's/doctorate), and frequency of recent public health inspections are key predictors of good knowledge and positive attitudes toward safety, this study provides a data-driven framework for targeted public health interventions. The knowledge-attitude gap identified, particularly in leadership roles where business owners and department heads were significantly less likely to demonstrate adequate knowledge than head chefs, highlights a systemic weakness. If these gaps are not addressed through specialized, training-specific programs with an emphasis on foodborne diseases and HACCP procedures, they could lead to large-scale outbreaks of foodborne illnesses. Ultimately, investment in personalized employee training fosters a strong food safety culture and contributes significantly to protecting public health and preventing foodborne illnesses.
